# Patient safety culture in Iranian teaching hospitals: baseline assessment, opportunities for improvement and benchmarking

**DOI:** 10.1186/s12913-022-07774-0

**Published:** 2022-03-26

**Authors:** Edris Kakemam, Ahmed Hassan Albelbeisi, Samane Davoodabadi, Masoud Ghafari, Zahra Dehghandar, Pouran Raeissi

**Affiliations:** 1 Tabriz Health Services Management Research Center, Tabriz University of Medical Sciences, Tabriz, Iran; 2 Medical Services Directorate, Gaza Strip, Palestine; 3 Department of Health Services Management, Health School, Arak University of Medical Sciences, Arak, Iran; 4 Deputy of Treatment, Alborz University of Medical Sciences, Karaj, Iran; 5 Department of Health Services Management, School of Health Management and Information Sciences, Iran University of Medical Sciences, Tehran, Iran

**Keywords:** Patient safety, Medical errors, Quality improvement, Benchmarking, Iran

## Abstract

**Background:**

Patient safety culture is an essential factor in determining the ability of hospitals to treat and reduce patient risks. Healthcare professionals, especially nurses, play an important role in patient safety because they are responsible for direct and ongoing patient care. Few studies in Iran examine the patient safety culture in Iranian teaching hospitals, particularly from the perspective of nursing staff. This research assessed patient safety culture in teaching hospitals in Iran from the nurses’ point of view and compared the outcomes with similar regional and global studies. Furthermore, the study identified the factors influencing patient safety culture and its association with outcomes.

**Methods:**

A cross-sectional study was accomplished in thirty-two teaching hospitals in five provinces of Iran. A total of 2295 nurses were chosen through convenience sampling. Collection data were done using the Hospital Survey of Patient Safety Culture (HOPSC) from October 2018 and September 2019. We analyzed the data using descriptive statistics, independent sample t-test, one-way ANOVA, and multiple linear regression analysis.

**Results:**

The results demonstrated the overall percentage of positive response rate for the HOPSC tool (36.4%). The average percentage of positive responses among all dimensions ranged from 27.1% in “Staffing” to 53.8% in “Teamwork across Hospital Units”. Benchmarking analysis shows that Iranian hospitals are equal or better performance than the benchmark on several composites compared to regional and global findings. The results of multiple linear regression analysis showed that the age, gender, total years of experience in nursing, work area or unit, work hours, and size of the hospital were significant predictors of the perceptions patient safety culture of nurses (*p* <  0.05).

**Conclusions:**

This is one of few studies that examine nurses’ perceptions of patient safety culture in public hospitals in Iran. Although the results of the present study showed that the results of Iran were at or better than the many composites in Jordan, Turkey, KSA, and the Philippines. The findings confirmed that all 12 dimensions can be considered as areas requiring improvement, and these results demonstrated that there was a severe shortage in patient safety culture among the included hospitals.

## Background

Patient safety is an international health concern affecting patients in various health care settings, patient safety as a health care discipline has emerged as a major concern due to the complexity of health care systems and the increase in unsafe care delivered to patients in various health services institutions [1, 2]. Patient safety is defined as “a framework of organized activities that creates cultures, processes, procedures, behaviors, technologies, and environments in health care that consistently and sustainably lower risks, reduce the occurrence of avoidable harm, make the error less likely and reduce its impact when it does occur” [3]. In the era of Universal Health Coverage (UHC), patient safety is a prerequisite of high-quality and safe healthcare services, thereby enabling a system to meet the needs for successful UHC [2]. The healthcare systems in developing countries are falling short of the adequate capacity and readiness for effective healthcare provision, which is coupled with shortages of medicines, medical equipment and laboratory tests, poor quality of care service, poor teamwork, and unsafe health care [4–7]. Reports indicate that undesirable events due to unsafe care are attributed to mortality and disability globally, despite the potential preventability of nearly half of these adverse events [8–10].

The main medical procedures that substantially contribute to unsafe care and harm to patients: are medications error, nosocomial infections, unsafe surgeries, injections procedures, and diagnostic errors [11, 12]. In the Eastern Mediterranean region states including Iran, approximately 80% of hospitalized patients are at risk of harm, about 62% of these adverse events are preventable, and medications errors are a significant problem [13]. In Iran, a review displayed that the prevalence of adverse events exists between 10 to 80% [14]. Another study displayed that about 70 % of participants’ professional nurses declared engaging in adverse events resulting in harm to patients [15]. Patient safety culture could be reported as comprehension of norms, principles, and standards regarding what is fundamental in an institution, what is supported and rewarded, and expected behaviors and attitudes related to patient safety. This structure mirrors the intangible parts of healthcare, influenced by the leadership, supervision, and feedback of professionals. Healthcare providers recognize that they have a pivotal role in closely following procedures. Therefore, they outline their activities by carrying out good practices and providing information for continuous improvement [16]. Then, any health organization must pay attention and understand its strengths and weaknesses in patient safety culture, it can especially help hospitals to identify current patient safety problems [17]. A healthcare system that has successfully created a patient safety culture is expected to translate this culture into remarkable actions that can lead to a reduction in adverse events and associated costs [18]. Measures taken to improve patient safety have demonstrated different levels of effectiveness [19]. Several previous studies among healthcare professionals have shown that higher levels of awareness of patient safety culture are associated with a lower occurrence of adverse events [20, 21]. Nurses play an important role in patient safety because they are responsible for direct and ongoing patient care [22] . Few studies in Iran examine the patient safety culture in Iranian teaching hospitals, particularly from a nursing staff perspective. Hence, this study aims to (1) measure patient safety culture in Iranian teaching hospitals from nursing staff perspective, (2) compare the patient safety culture with similar regional and global studies, and (3) examine the relationship between patient safety culture and patient safety grade and frequency of events reported, and (4) to identify the factors influencing patient safety culture in Iranian hospitals.

## Methods

### Study design

We conducted a survey using a cross-sectional design. To represent teaching hospitals across the country, the survey was conducted in 32 teaching hospitals grouped into five provincial centers. Since the organizational framework and management of the general and education hospitals are similar in every public hospital within Iran and managed through the same regulations of the ministry of health, the study can be considered partially representative of the entire hospitals.

### Population and sampling

The target group included all nursing staff working in the included teaching hospitals (about 9000). The participant must meet the following points (1) a clinical nurse; (2) working for 12 months in hospital units to ensure they are familiar with units’ regulations; and (3) willingness to participate during the data collection period. On the other hand, nurses are excluded in the following cases: are practical nurses with less than a bachelor’s certificate. Furthermore, nurses with less than 12 months of work in hospital units, and nonclinical nurses. By using the convenience sampling method, 3500 questionnaires were distributed among nurses.

### Instrument

Data collection was conducted using the Hospital Survey of Patient Safety Culture (HSOPSC). This instrument was established by the Agency for Healthcare Research and Quality and is developed to measure patient safety culture in healthcare facilities [3]. It is a self-administration instrument with 12 dimensions and one outcome that can be utilized to assess patient safety culture for the entire hospital. Moghri et al. (2012) have translated and utilized this survey into Persian and reviewed the translation validity [23]. The reliability of this tool was reported between 0.57 and 0.8 [23]. However, to ensure reliability, Cronbach’s alpha was calculated for all dimensions of the questionnaire using the data of the current study (Table 2). In addition, the survey included some demographic information such as age, sex, education level, work experience in nursing, work department, hours worked per week, and size of the hospital.

### Data collection

Due to the heavy workload of nurses and the low response rate at the beginning of the study period, data collection was conducted between October 2018 and September 2019. Data was collected using printed questionnaires. The first page of the questionnaire included a cover letter briefly explaining the purpose of the study. Informed consent is signed before the participant completes the questionnaire, voluntary and confidential participation is guaranteed. Due to the heavy workload of the nurses, the researchers left the questionnaires with them. The researchers made regular visits to the departments during the workweek to gather the completed questionnaires.

### Data analysis

Incomplete questionnaires and the questionnaires containing the same answer to all the questions in the survey were excluded. The HSOPSC includes both positive and negative worded items scored via a five-point scale that reflects agreement or frequency of occurrence. The tool includes 44 items, 42 of which measure 12 patient safety culture composites (two of which are patient safety culture outcomes). The two outcome variables are patient safety grade and the number of events.

We used the descriptive statistics indicators (frequencies and percentages) to describe the sociodemographic characteristics of the participants. Based on the user’s manual of the HSOPSC, the frequency count of positive responses in each item was conducted to determine the item’s percent positive scores. The percent positive score composite scores were computed by obtaining the average of the percent positive responses on all items included in the composite [(number of positive responses/total number of respondents on the item) × 100%]. The composites that were rated positively by 70% or more of the respondents were regarded as strengths, whereas those with a score of below 70% were regarded as weaknesses and require to improve [24].

An independent sample t-test and One-way ANOVA were used to compare the score difference. A multiple linear regression analysis was used to determine the predictors. The model utilized the overall mean as the dependent variable and the sociodemographic characteristics of nurses as the predictor variables. All tests were conducted at 0.05 level of significance.

Results from these hospitals were also benchmarked against similar initiatives in Jordan [25], Turkey [26], Saudi Arabia (KSA) [27], and the Philippines [28]. Comparison to the benchmark value was done using the below formula:

$$\mathrm{Percentage}\ \left(\%\right)\ \mathrm{Distance}\ \mathrm{from}\ \mathrm{benchmark}=\left(\mathrm{benchmark}\ \mathrm{value}-\mathrm{hospital}\left(\mathrm{s}\right)\ \mathrm{result}/\mathrm{benchmark}\ \mathrm{value}\right)\ast 100$$

Categories of achievement were determined by the value of Percentage (%) distance from benchmark as follows:

- Values < 10% were categorized as Meets or better than the benchmark (  ). Values below zero (0) indicate that the benchmark value is lower than the hospitals result thus giving a result of “meet or better than benchmark”.
- Values between [10–50%] were categorized as Deviates slightly from the benchmark (■).
- Values exceeding 50% were categorized as Major deviation from the benchmark (  ).

All data analyses were performed using SPSS 22.0 (IBM Corp., Armonk, NY, USA).

## Results

The sociodemographic characteristics of the participants are presented in Table 1. Of the 3500 questionnaires distributed, a total of 2410 nurses returned this survey. However, 115 questionnaires that were incomplete or contained the same answers were excluded. The response rate was 65.6%. Nearly 80 % of the respondents were female, more than half of them are married (51.8%), and with a bachelor of science in nursing degree (77.4%). The mean age was 34.14 years (SD = 7.07) years. Almost half of them are between the ages of 31 to 40 years (46.6%). Most of the participants (76.3%) worked between 40 and 60 h per week. The average years of experience was 8.96 (SD = 6.77).

**Table 1:** Participants’ sociodemographic characteristics and means of patient safety culture

Variables	Number	%	Patient safety culture	*P*
			Mean (SD)	
**Gender**				< 0.001
Male	468	20.4	3.09 (0.33)	
Female	1827	79.6	3.19 (0.38)	
**Age**				< 0.001
Below 30 years	840	36.6	3.15 (0.34)	
30–40 years	1070	46.6	3.15 (0.37)	
Above 40 years	385	16.8	3.27 (0.42)	
**Education level**				0.191
Bachelor’s Degree	1777	77.4	3.17 (0.37)	
Masters or PhD Degree	518	22.6	3.15 (0.35)	
**Work experience in nursing (years)**				< 0.001
1–5	922	40.2	3.13 (0.34)	
6–10	615	26.8	3.14 (0.37)	
* > 10*	758	33.0	3.24 (0.39)	
**Work area**				< 0.001
Critical care units	533	23.2	3.22 (0.37)	
Emergency department	458	20.0	3.07 (0.31)	
General wards	1304	56.8	3.18 (0.38)	
**Size of hospital (number of bed)**				< 0.001
< 200	856	37.3	3.21 (0.38)	
200–500	1040	45.3	3.13 (0.36)	
> 500	399	17.3	3.18 (0.36)	
**Hours worked per week**				< 0.001
< 40 h	422	18.4	3.11 (0.35)	
40–60	1729	76.3	3.19 (0.38)	
> 60 h	144	6.3	3.07 (0.31)	
**Number of events reported (during the past 1 year)**				< 0.001
No event reports	1014	45.4	3.10 (0.33)	
1–5	1058	46.1	3.19 (0.36)	
> 5	196	8.5	3.42 (0.45)	
**Patient Safety Grade**				< 0.001
Excellent	191	8.3	3.40 (0.50)	
Very good	1014	44.2	3.23 (0.36)	
Acceptable	884	38.5	3.09 (0.29)	
Poor	179	7.8	3.01 (0.36)	
Failing	27	1.2	2.72 (0.51)	

*SD *Standard deviation

### Strengths and areas for improvement

Strength area (the percentage of positive rating above 70%), Area requiring improvement (the percentage of positive rating below 70%). All twelve subscales can be considered areas requiring improvement. The largest area of strength highlighted by the responses was the item related to “It is just by chance that more serious mistakes do not happen around here” where the positive response rate was 71.1%. Other areas of strength were revealed within the subscale of Supervisor/Manager Expectations & Actions Promoting Patient Safety whereby the item on emphasis on patient safety issues by the manager received 61.0% positive responses. Staffing-related areas require improvement. Respondents indicated that hospital staff did not try to do too much, too quickly when the work is in “crisis mode” (19.5% positive response). As for the subscale on Communication Openness, 21.0% of employees were stated that did not feel free to question the decisions or actions of those with more authority. The other items are listed in Table 2.

**Table 2:** Cronbach’s alpha and distribution of positive responses and scores for survey subscales and items

Subscales and survey items	Average% positive response^a^	Mean (SD)
***1. Overall Perception of Safety (Cronbach’s α = 0.76)***	***47.5***	**3.40 (.57)**
1.1. It is just by chance that more serious mistakes do not happen around here (R)^b^	71.1	3.95(.93)
1.2. Patient safety is never sacrificed to get more work done	46.8	3.35(1.11)
1.3. We have patient safety problems in this unit (R)	27.0	2.99(.96)
1.4. Our policies and procedures and systems are effective in preventing errors	44.9	3.32(.96)
***2. Supervisor/Manager Expectations & Actions Promoting Patient Safety (Cronbach’s α = 0.81)***	***44.3***	***3.27 (.71)***
2.1. My supervisor/manager says a good word when he/she sees a job done according to established patient safety procedures	28.2	2.85(1.12)
2.2. My supervisor/manager seriously considers staff suggestions for improving patient safety	36.4	3.12(1.02)
2.3. Whenever pressure builds up, my supervisor/manager wants us to work faster, even if it means taking shortcuts (R)	51.7	3.45(1.15)
2.4. My supervisor/manager overlooks patient safety problems that happen over and over (R)	61.0	3.67(1.14)
***3. Organizational learning and Continuous Improvement (Cronbach’s α = 0.69)***	***42.7***	***3.31 (.73)***
3.1. We are actively doing things to improve patient safety	49.0	3.43(.98)
3.2. Mistake have led to positive changes here	40.4	3.26(.93)
3.3. After we make changes to improve patient safety, we evaluate their effectiveness	38.6	3.24(.95)
***4. Teamwork within units (Cronbach’s α = 0.75)***	***43.8***	***3.27 (.78)***
4.1. Staff support one another in this unit	39.4	3.23(.98)
4.2 When a lot of work needs to be done quickly, we work together as a team to get the work done	49.9	3.41(1.02)
4.3. In this unit, people treat each other with respect	54.6	3.46(1.02)
4.4. When members of this unit get really busy, other members of the same unit help out	31.4	2.96 (1.09)
***5. Non-punitive Response to Error (Cronbach’s α = 0.69)***	***27.4***	***2.86 (.82)***
5.1. Staff feel like their mistakes are held against them (R)	25.3	2.85(1.02)
5.2. When an event is reported, it feels like the person is being written up, not the problem (R)	30.9	2.92(1.09)
5.3. Staff worry that mistakes they make are kept in their personnel file (R)	25.9	2.80(1.09)
***6. Staffing (Cronbach’s α = 0.82)***	***27.1***	***2.80 (.71)***
6.1 We have enough staff to handle the workload	25.2	2.64(1.18)
6.2. Staff in this unit work longer hours than is best for patient care (R)	28.8	2.83(1.17)
6.3. We use agency/temporary staff than is best for patient care (R)	35.0	3.06(1.10)
6.4. When the work is in “crisis mode” we try to do too much, too quickly (R)	19.5	2.68(1.02)
***7. Hospital Management Support for Patient Safety (Cronbach’s α = 0.71)***	***35.8***	***3.16 (.65)***
7.1. Hospital management provides a work climate that promotes patient safety	43.0	3.27(.95)
7.2. The actions of hospital management show that patient safety is a top priority	35.8	3.10(1.06)
7.3. Hospital management seems interested in patient safety only after an adverse event happens (R)	34.5	3.10(1.00)
***8. Teamwork Across Hospital Units (Cronbach’s α = 0.74)***	***53.8***	***3.14 (.59)***
8.1. There is good cooperation among hospital units that need to work together	34.1	3.09(1.01)
8.2. Hospital units work well together to provide the best care for patients	33.6	3.09(.97)
8.3. Hospital units do not coordinate well with each other and this might affect patient care (R)	41.4	3.26(1.02)
8.4. It is often not easy to work with staff from other hospital units (R)	34.0	3.13(.95)
***9. Hospital Handoffs & Transitions (Cronbach’s α = 0.79)***	***45.4***	***3.37 (.82)***
9.1. Things “fall between the cracks”, i.e., things might go uncontrolled and get lost when transferring patients from one unit to another (R)	40.5	3.29(.97)
9.2. Important patient care information is often lost during shift changes (R)	50.7	3.47(1.04)
9.3. Problems often occur in the exchange of information across hospital units (R)	41.4	3.31(.98)
9.4. Shift changes are problematic for patients in this hospital (R)	49.1	3.39(1.18)
**10. Communication Openness (Cronbach’s α = 0.80)**	***34.1***	***3.02 (.62)***
10.1. Staff will freely speak up if they see something that may negatively affect patient care	39.1	3.17(1.00)
10.2. Staff feel free to question the decisions or actions of those with more authority	21.0	2.62(1.05)
10.3. Staff are afraid to ask questions when something does not feel right (R)	42.1	3.27(.99)
**11. Feedback and Communications About Error (Cronbach’s** ***α* = 0.73)**	***41.1***	***3.24 (.74)***
11.1. We are given feedback about changes put into place based on event reports	40.7	3.24(.97)
11.2. We are informed about errors that happen in this unit	44.5	3.28(.99)
11.3. In this unit, we discuss ways to prevent errors from happening again	38.2	3.20(.98)
**12. Frequency of events reported (Cronbach’s α = 0.90)**	***37.7***	3.10 (.73)
12.1. When a mistake is made, but is caught and corrected affecting the patient, how often is this reported?	46.8	3.28(1.08)
12.2. When a mistake is made, but has no potential to harm the patient, how often is this reported?	31.2	3.02(.95)
12.3. When a mistake is made that could harm the patient, but does not, how often is this reported?	35.0	3.01(1.05)
***Average patient safety culture percentage across all composites***	***36.4***	***3.17 (0.37)***

^a^The composite-level percentage of positive responses was calculated using the following formula: (number of positive responses to the items in the composite/ total number of responses to the items (positive, neutral, and negative) in the composite (excluding missing responses)) *100, ^b^ Negatively worded items were coded reversed

In terms of the patient safety grade, 52.5% of the participants rated their hospital as excellent/very good, whereas 38.5 and 9.0% rated it as acceptable and poor/failing, respectively. In addition, 45.4% of respondents did not report any event in the past year, whereas 46.1% made one to five event reports in the past year. Only 8.5% made more than five event reports in the past year.

### Association between patient safety grade and number of events with subscales

The associations between patient safety culture subscales**,** patient safety grade, and the number of events are presented in Table 3. Nurses who rated “Excellent/Very Good” patient safety grades had significantly the highest mean scores for patient safety subscales. Furthermore, the number of events reported was significantly associated with all the patient safety subscales. Participants who reported more errors had higher perceptions of all subscales of patient safety culture.

**Table 3:** Comparison between patient safety grade and number of events reported with patient safety culture composite scores (Composites scored range from 1 to 5)

Subscales of PSC	Patient Safety Grade			p-value*	Number of Events Reported			p-value*
	Poor or Failing	Acceptable	Excellent/ Very Good		No event reports	1 to 5 events reports	> 5 events reported	
	Mean (SD)	Mean (SD)	Mean (SD)		Mean (SD)	Mean (SD)	Mean (SD)	
Teamwork within units	3.05 (0.91)	3.18 (0.72)	3.37 (0.72)	*<.001 (b,c)*	3.16 (0.77)	3.31 (0.76)	3.60 (0.80)	*<.001 (a,b,c)*
Supervisor/manager expectations and actions promoting patient safety	3.09 (0.76)	3.18 (0.67)	3.38 (0.72)	*<.001 (b,c)*	3.18 (0.70)	3.31 (0.72)	3.56 (0.65)	*<.001 (a,b,c)*
Organizational learning-continuous improvement	3.14 (0.85)	3.24 (0.64)	3.39 (0.76)	*<.001 (b,c)*	3.24 (0.70)	3.34 (0.75)	3.59 (0.74)	*<.001 (a,b,c)*
Management support for patient safety	2.79 (0.79)	3.04 (0.54)	3.31 (0.66)	*<.001 (a,b,c)*	3.07 (0.63)	3.18 (0.64)	3.49 (0.74)	*<.001 (a,b,c)*
Feedback and communication about error	2.95 (0.83)	3.14 (0.66)	3.36 (0.75)	*<.001 (a,b,c)*	3.14 (0.71)	3.27 (0.74)	3.63 (0.71)	*<.001 (a,b,c)*
Communication openness	2.81 (0.67)	2.97 (0.59)	3.09 (0.62)	*<.001 (a,b,c)*	2.94 (0.61)	3.04 (0.59)	3.37 (0.71)	*<.001 (a,b,c)*
Teamwork across hospital units	2.93 (0.54)	3.05 (0.52)	3.24 (0.62)	*<.001 (a,b,c)*	3.07 (0.57)	3.16 (0.57)	3.43 (0.69)	*<.001 (a,b,c)*
Staffing	2.72 (0.84)	2.76 (0.69)	2.84 (0.70)	*.012 (c)*	2.80 (0.69)	2.78 (0.72)	2.89 (0.75)	*.150*
Hospital handoffs and transitions	3.22 (0.73)	3.26 (0.68)	3.47 (0.91)	*<.001 (b,c)*	3.29 (0.81)	3.39 (0.82)	3.66 (0.80)	*<.001 (a,b,c)*
Non-punitive response to error	2.72 (0.98)	2.81 (0.79)	2.92 (0.82)	*.001 (b,c)*	2.81 (0.80)	2.86 (0.83)	3.08 (0.89)	*<.001 (b,c)*
Patient Safety Grade a. Significant difference between “Poor or Failing” and “Acceptable” b. Significant difference between “Poor or Failing” and “Excellent/Very Good” c. Significant difference between “Acceptable” and “Excellent/Very Good”			Number of Events Reported a. Significant difference between “No events reported” and “1 to 5 events reported” b. Significant difference between “No events reported” and “> 5 events reported” c. Significant difference between “1 to 5 events reported” and “> 5 events reported”					

* One-way ANOVA

### Factors influencing nurses’ perception towards patient safety culture

The results of multiple linear regression analysis to examine the factors of patient safety culture shows in Table 4. The multiple linear regression model with all (eight) predictors was statistically significant, F = 10.44, *p* < .001, and it accounted for 32% of the variance in the nurses’ perceptions (R^2^ = .35; adjusted R^2^ = .32). The results showed that the age, gender, marital status, tenure in nursing, work area or unit, work hours, and size of the hospital were significant predictors of the perceived patient safety culture of nurses.

**Table 4:** Predictors of nurses’ perceptions of patient safety culture in Iranian hospitals

Variables	B	95% CI^a^		*P*-value
**Age** (*Reference group: Above 40 years*)				
Below 30 years	−.040	−.103	.024	.219
30–40 years	−.077	−.125	−.029	*.002*
**Gender** (*Reference group: female*)	−.084	−.120	−.047	*<.001*
**Work experience in nursing(***Reference group: > 10***)**				
1–5	−.079	−.133	−.025	*.004*
6–10	−.061	−.105	−.017	*.006*
**Education level (***Reference group: Masters or PhD Degree***)**	.033	−.003	.069	.072
**Work Area (***Reference group: General wards***)**				
Critical care units	.035	−.001	.071	.057
Emergency department	−.091	−.130	−.052	<.001
**Hours worked per week (***Reference group: < 40 h***)**				
40–60	.007	−.061	.075	.843
> 60 h	.070	.008	.132	*.028*
**Size of hospital (***Reference group: > 500 bed***)**				
< 200	.021	−.023	.064	.354
200–500	−.058	−.100	−.015	.007

^a^Confidence Interval

Regression analysis revealed that male respondents reported a lower perception of patient safety than female respondents (β = −.084, *P* < .001). Older nurses had a better perception of patient safety than younger nurses (β = −.077, *P* = .002). In addition, as total years of experience of nurses increased, overall perception of patient safety progressively increased (β = −.079, *P* = .004; β = − 0.061; *P* = .006). Respondents working in the general wards or units of the hospital had better perceptions of patient safety than those working in the emergency departments (β = −.091, *P* < 0.001). Regarding working hours per week, participants who worked less than 40 h weekly had a significantly lower perception of patient safety culture compared with nurses who worked more than 60 h weekly (β = .070, *P* = .028). Nurses working in the medium hospitals (200–500 beds) had lower perceptions of patients’ safety culture compared with nurses who worked in large hospitals (> 500 beds) (β = −.058, *P* = .007).

### Benchmarking

In Table 5, the results of the present research were compared with similar studies that were conducted in Jordan [25], Turkey [26], KSA [27], and the Philippines [28]. These studies were selected because they are the most recent (2015–2018) and reflect the results of national studies. Iran results equal or better performance than the Jordan benchmark for the two subscales: “Teamwork across hospital units” and “Non-punitive response to error”.

**Table 5:**
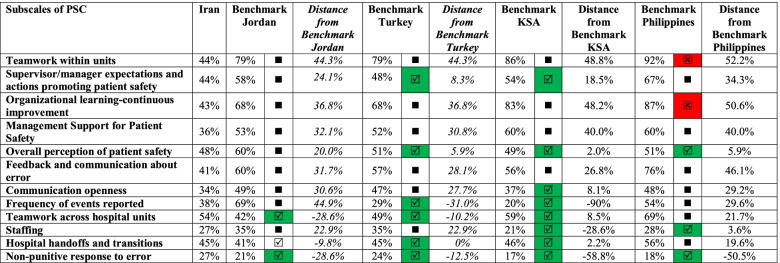
Benchmarking Percent Positive on Survey Composites from Iran against those in Jordan, Turkey, KSA, Philippines

Result meets or better than the benchmark (results within 10% of benchmark)

■ Deviates slightly from benchmark (results 10–50% from benchmark)

Deviation from benchmark (results exceeding 50% difference with benchmark)

Iran had subscales scores that were equal or better performance than the benchmark for Turkey for six of the subscales: “Supervisor/manager expectations and actions promoting patient safety”, “Overall perception of patient safety”, “Frequency of events reported”, “Teamwork across hospital units”, “Hospital handoffs and transitions”, and “Non-punitive response to error”.

When compared with KSA, Iran’s findings equal or better performance than the benchmark for eight of the subscales: “Supervisor/manager expectations and actions promoting patient safety”, “Overall perception of patient safety”, “Communication openness”, “Frequency of events reported”, “Teamwork across hospital units”, “Staffing, Hospital handoffs, and transitions”, “Non-punitive response to error”.

As compared to the Philippines, teaching hospitals were found to meet or exceed benchmarks for subscales pertaining to “Teamwork within units”, “Organizational learning-continuous improvement”, “Overall perception of patient safety”, “Staffing”, and “Non-punitive response to error”.

## Discussion

This is one of few studies that examine nurses’ perceptions of patient safety culture in teaching hospitals in Iran and compare the patient safety culture with similar regional and global studies. Although the results of our study showed that the results of Iran were equal or better performance than the Jordan benchmark for two subscales, were equal or better performance than the benchmark for Turkey for six of the subscales, were equal or better performance than the benchmark for KSA for eight of the subscales, and were equal or better performance than the Philippine benchmark for the three subscales, the findings confirmed that all areas of patient safety culture still require further work. There are two major deviations from benchmarks (Organizational learning-continuous improvement and Teamwork within units). More areas of minor deviation show that additional consideration is required to continually improve future performance. Compared to other states in the region displayed that the Iranian public hospitals are much better on some subscales. For instance, Non-punitive response to error had a percent positive score of 27% while it scored 21% in Jordan [25], 24% in Turkey [26], 17% in KSA [27], and 18% in the Philippines [28].

The results for the overall percentage of positive response rate for the HOPSC tool (36.4%), lower than several previous studies carried out in LMICs (e.g. Ethiopia (46.7%) [29], Lebanon (61.5%) [30], and Palestine(54.5%) [31]), and lower than several studies conducted in high-income countries (e.g. the KSA (61%) [32], Netherlands (52.2%) [33], and the USA (65%) [34]). The average percentage of positive responses among all dimensions was between 27.1% in “staffing” to 53.8% in “Teamwork across Hospital Units”.

“Staffing” was the lowest among all dimensions, this could reflect the nurses’ feeling that the number of nurses was not enough to achieve patient safety due to the workload. Inadequate staffing levels have been shown as one of the reasons why patient safety is difficult to achieve in LMICs in previous studies [29, 30, 35]. In contrast, the ‘staffing’ dimension rated higher in high-income countries [33, 34]. These differences may be due to the higher number of nurses in high-income countries compared to low-and middle-income countries, for example, in Iran, there is a shortage of nurses around 130,000, and there is and only 1.3 nurses per 1000 people, compared with the Organization for Economic Co-operation and Development average of 7.4 per 1000 people [36–38]”. Teamwork Across Hospital Units” was the highest among all dimensions, this result was consistent with a previous systematic review conducted in Iran (2015) which showed “Teamwork Across Hospital Units” received the highest score with (67.4%) [39]. All 12 dimensions can be considered as areas requiring improvement, and these results demonstrated that there was a severe shortage in patient safety culture among the included hospitals. Our study results are consistent with studies conducted in low-middle- and high-income countries [29, 40, 41].

In terms of the patient safety grade, 52.5% of the participants rated their hospitals as excellent/very good, whereas 38.5 and 9.0% rated it as acceptable and poor/failing, respectively. A previous study conducted in Iran demonstrated that 41% of respondents evaluated their hospitals as excellent/very good [39]. A study in Lebanon conducted among hospitals staff showed that 70% of participants evaluated their hospitals as excellent/very good [30]. In Ethiopia, 47.6% of nurses evaluated their hospitals as excellent/very good, and 52.4% as acceptable and poor/failing [29]. In addition, 45.4% of respondents did not report any event in the past year, whereas 46.1% made one to five incident reports in the past year. Only 8.5% made more than five incident reports in the past year. In Ethiopia, 68% of nurses made at least one event report in the past year, compared with 57% in the KSA, 41% in Lebanon, 45% in the USA, and 47% in Palestine [30–32, 34].

Nurses who rated “Excellent/Very Good” patient safety grades had significantly the highest mean scores for patient safety composites. Furthermore, the number of events reported was significantly associated with all the patient safety composites. Participants who reported more errors had higher perceptions of all composites of patient safety culture. The result is in agreement with prior studies that have revealed an association between safety culture and events reporting [42, 43]. In contrast, the Ethiopian study did not demonstrate an association between safety culture and events reporting [29].

The results displayed that the age, gender, marital status, period in nursing, work department or unit, work hours, and size of the hospital were significantly predictive of patient safety culture for nurses. Male nurses showed lower perceptions compared with female nurses. Older participants had lower perceptions of patient safety. The higher the total number of years of experience and age, the better the nurse’s perception of patient safety culture Nurses working in emergency units had better perceptions of patient safety than nurses in other units. A study conducted in Iran in three private hospitals and three public hospitals showed that the gender, age groups, department of work, and work experience were not predictive of perceived safety culture, in contrast, significant differences were found between patient safety culture score and type of hospital and shift work, the result of study confirmed that patient safety score in public hospitals was higher than private hospitals and also higher among shift work staff than those were not [44]. In KSA, a previous study showed that nurses working in emergency units had better perceptions of patient safety than those working in other departments, In contrast to the results of our study, nurses with less than 1 year of experience have better perceptions of patient safety [27]. Several previous studies showed that the higher total years of experience, the better the nurse’s perception of patient safety culture [45, 46].

In addition, nurses who worked more hours per week had lower perceptions of patients’ safety culture compared with nurses who worked fewer hours per week. Working in a medium hospital (200–500 beds) decreased nurses’ perceptions of patients’ safety culture compared with nurses who worked in large hospitals (> 500 beds). A study conducted in KSA showed that nurses who work between 40 and 59 h per week had better perceptions of patient safety culture than those working for < 40 or ≥ 60 per week [47]. Studies conducted in Lebanon, Kuwait showed that the small hospitals were found to have a more positive perception of patient safety culture and higher scores compared to large hospitals [30, 41].

## Limitations

The main limitations of this study, including the use of a convenience sampling method, which could lead to selection bias. Besides, other healthcare professionals, such as physicians, laboratory technicians, and paramedics, were not included in this study. In addition, the length of collection data is 12 months. Finally, the study is dependent on a questionnaire based on self-reported that could lead to bias (recall and social desirability).

## Conclusions

This is one of few studies that examine nurses’ perceptions of patient safety culture in public hospitals in Iran. Although the findings of our research showed that the results of Iran were at or better than the many composites in Jordan, Turkey, KSA, and the Philippines. The findings confirmed that all 12 dimensions can be considered as areas requiring improvement, and these results demonstrated that there was a severe shortage in patient safety culture among the included hospitals. These findings are of paramount importance in the Iranian context, such assessments can provide worthy data to hospital managers on how work performance has shifted as a result of quality policies. Hospital administrators should make more attention to examining these issues, to improve reporting, the overall perception of patient safety, and patient safety grades.

## Data Availability

The datasets used and/or analyzed during the current study are available from the corresponding author on reasonable request.

## Abbreviations

UHC: Universal Health Coverage
LMICs: Low and middle-income countries
HOPSC: Hospital Survey of Patient Safety Culture
KSA: Saudi Arabia
